# Abnormal [18F]FDG PET/MRI findings in paraspinal structures of patients with suspected cerebrospinal fluid leak

**DOI:** 10.1038/s41598-021-95056-w

**Published:** 2021-08-05

**Authors:** Daehyun Yoon, Peter William Cipriano, Ryan Penticuff, Jessa Ballesteros Castillo, Yingding Xu, Ian Richard Carroll, Sandip Biswal

**Affiliations:** 1 Department of Radiology, Stanford University, 300 Pasteur Drive, Stanford, CA 94305 USA; 2 Department of Radiology, UC San Diego School of Medicine, La Jolla, CA USA; 3 Newport Harbor Radiology Associates, Irvine, CA USA; 4 Department of Anesthesia Perioperative and Pain Medicine, Stanford University, 300 Pasteur Drive, Stanford, CA 94305 USA

**Keywords:** Magnetic resonance imaging, Positron-emission tomography, Diagnostic markers, Biomedical engineering

## Abstract

A combination of magnetic resonance imaging (MRI), computed tomography (CT), and radionuclide cisternography are typically used to locate a cerebrospinal fluid (CSF) leak. However, the site of leakage cannot be determined, making treatment more difficult. Therefore, more sensitive imaging tools are needed. A whole-body [18F]fluorodeoxyglucose (FDG) positron emission tomography (PET)/MRI was conducted on six patients with suspected CSF leak and the resulting images were reviewed in comparison with those from six healthy controls. Paraspinal regions of focally increased uptake of [18F]FDG were quantified using maximum standardized uptake values (SUV_max_) and compared to the SUV_max_ of corresponding regions in the healthy controls. All six patients with suspected CSF leak showed paraspinal regions of significantly greater [18F]FDG uptake compared to the corresponding areas in controls (*P* < 0.05). Two patients treated with local injections (epidural blood patches and/or epidural fibrin patches) on the site of abnormal PET/MRI findings reported temporary but significant improvement in symptoms. Our results suggest [18F]FDG PET/MRI is sensitive to abnormalities potentially due to suspected CSF leak, which are not necessarily visible on conventional MRI alone or by the standard-of-care imaging methods.

## Introduction

Cerebrospinal fluid (CSF) leak is a condition caused by drainage of CSF from the brain or the spinal cord through a hole in their surrounding structures, such as the skull and the dura. The prevalence rate of the CSF leak is estimated to be 1 in 50,000 individuals, but this relatively uncommon occurrence may be due to underdiagnosis^1^. The most characteristic symptom of a CSF leak is a persistent headache that worsens when upright and improves with recumbency (orthostatic headaches)^2^. When chronic, CSF leaks may present as “second half of the day headache” with patients consistently better in the morning after prolonged nocturnal recumbency followed by worsening symptoms as the day progresses with prolonged upright time^3^. Other symptoms include nausea, memory difficulties, cranial nerve dysfunction, neck and interscapular pain^4,5^. Hereditary disorders of connective tissue such as Marfan Syndrome, Ehlers-Danlos Syndrome, Neurofibromatosis type 1, and Polycystic kidney disease have been associated with spinal CSF leaks, suggesting these and other associated disorders of connective tissue may lead to abnormally vulnerable dura prone to CSF leak^6^. Among those who can identify a precipitating event, atraumatic activities such as stretching, or Valsalva maneuver are commonly involved^7–9^. Traumatic head/neck injury such as whiplash as well as iatrogenic dural tear during surgical interventions including lumbar puncture, durotomy, and skull base surgery are also important causes^10–12^. However, two thirds of patients with a spontaneous CSF leak can identify no precipitating events. The most common site of leak is along the spinal cord^13^. If the site of CSF leak is located, it is treated most commonly with an epidural injection of autologous blood (epidural blood patch) or an epidural fibrin patch, or surgery, depending on the case^14^.

Unfortunately, no one diagnostic test can consistently exclude the diagnosis of CSF leak or identify the site(s) of leakage^15^. Misdiagnosis is common, preventing proper treatment as the symptoms are often mistaken for those of other diseases, such as postural orthostatic tachycardia syndrome or new daily persistent headache^14,16^. The underlying sensitivity of magnetic resonance imaging (MRI) and computed tomography (CT) myelography remains unknown. While these two imaging modalities often are consistent in retrospective comparison^17^, this may reflect the common clinical scenario of using invasive CT myelography much more frequently in patients with positive MRI than in patients with negative MRI—attempting to localize the source of leak suggested by findings seen on the subset of positive MRI. In contrast, prospective studies of patients with orthostatic headaches suggest a potentially much lower sensitivity. In one study of 100 patients with orthostatic headaches suspected of having CSF leaks from a consortium of 11 hospitals in Japan, 70 had axial T2 spine MRI, finding 14 leaks, while 86 had MR myelograms, where only 3 leaks were noted^18^. For CT myelography, cases with slow or intermittent CSF leakage are very difficult to identify due to the insufficient contrast and resolution between CSF and background^19^. Previous studies estimate that 46–55% of these cases present no detectable signs of CSF leak on CT myelogram^20,21^. Thus, better non-invasive imaging techniques for detecting and localizing spinal CSF leaks are needed.

In this report, we introduce the use of simultaneous [18F]fluorodeoxyglucose (FDG) positron emission tomography (PET)/MRI to locate CSF leaks. We hypothesized that [18F]FDG PET, if coupled with the high spatial and contrast resolution of MRI, could accurately identify inflammation around the sites of CSF leak due to its pro-inflammatory nature^22^. We recently presented initial findings from our study^23^, and here we describe in detail our early experience of [18F]FDG PET/MRI of patients with chronic symptoms of suspected CSF leaks in comparison with healthy controls.

## Results

### Abnormal [18F]FDG PET/MRI findings

In all six patients, we observed sites of abnormally increased focal uptake of [18F]FDG in paraspinal structures at various levels of the spine (cervical, thoracic, and lumbar) as summarized in Table 1. However, only three patients showed MRI abnormalities (abnormal signal increase on T2-weighted contrast), which were in the same corresponding region of the PET abnormalities (Table 1). Abnormal [18F]FDG uptake was found on paraspinal muscles in 3 patients, interspinous ligament in 2 patients, osseous tissue in 3 patients, and fluid collection in one patient. Except one patient case, all other cases presented abnormalities at multiple spine levels.

**Table 1:** Summary of abnormal paraspinal findings from [18F]FDG PET/MRI of 6 patients with suspected CSF leak.

Patient	Sites of increased [18F]FDG uptake	MRI abnormalities
Pt. 1	Interspinous ligaments from L4-5	T2 hyperintensity of paraspinal muscles and interspinous ligament at L4-5
Pt. 2	Interspinous ligaments from L1-5	None
Pt. 3	Intra/extrathecal space extending into neuroforamina at C2 Paraspinal muscles at C6 (bilateral), T6-7 (right), T8 (right)	None
Pt. 4	Interspinous ligaments from L2-3 Left neuroforamina from L3-4 Right neuroforamina/facet joints from L4-5	None
Pt. 5	Interspinous ligaments, paraspinal muscles, and spinous processes from L2-3	Large focal T2 hyperintensity of the paraspinal soft tissues at L2-3
Pt. 6	Multiple fluid collections from T10-L1 Spinous process and neuroforamen at T10	Multiple fluid collections from T10-L1 Bilateral pars defects at L5

The [18F]FDG SUV_max_ in lesions from the patient group showed a higher mean value than the corresponding areas from the control group in all tissue types (Table 2). The SUV_max_ of the abnormal lesions in the patient group ranged from 1.148 to 6.565 while the SUV_max_ of the corresponding tissues in the controls ranged from 0.416 to 2.893. *P*-values from the Mann–Whitney U-test comparing the SUV_max_ between the patient and control groups were less than 0.05 in all tissue types, indicating a significant difference of the [18F]FDG uptake between the two groups.

**Table 2:** SUV_max_ (mean ± standard deviation) from detected lesions of the patient group and corresponding regions of the control group for different tissue types.

Tissue type	Patients	Controls	*P*-values
Paraspinal muscle	2.26 ± 0.78	1.00 ± 0.48	0.0017
Interspinous ligament	2.44 ± 0.73	1.07 ± 0.25	0.000024
Osseous tissue	3.53 ± 1.37	0.98 ± 0.19	0.0018
Neuroforamen	2.50 ± 1.37	1.20 ± 0.23	0.033
Fluid collection	5.30 ± 0.96	1.10 ± 0.32	0.000028

**Figure 1:**
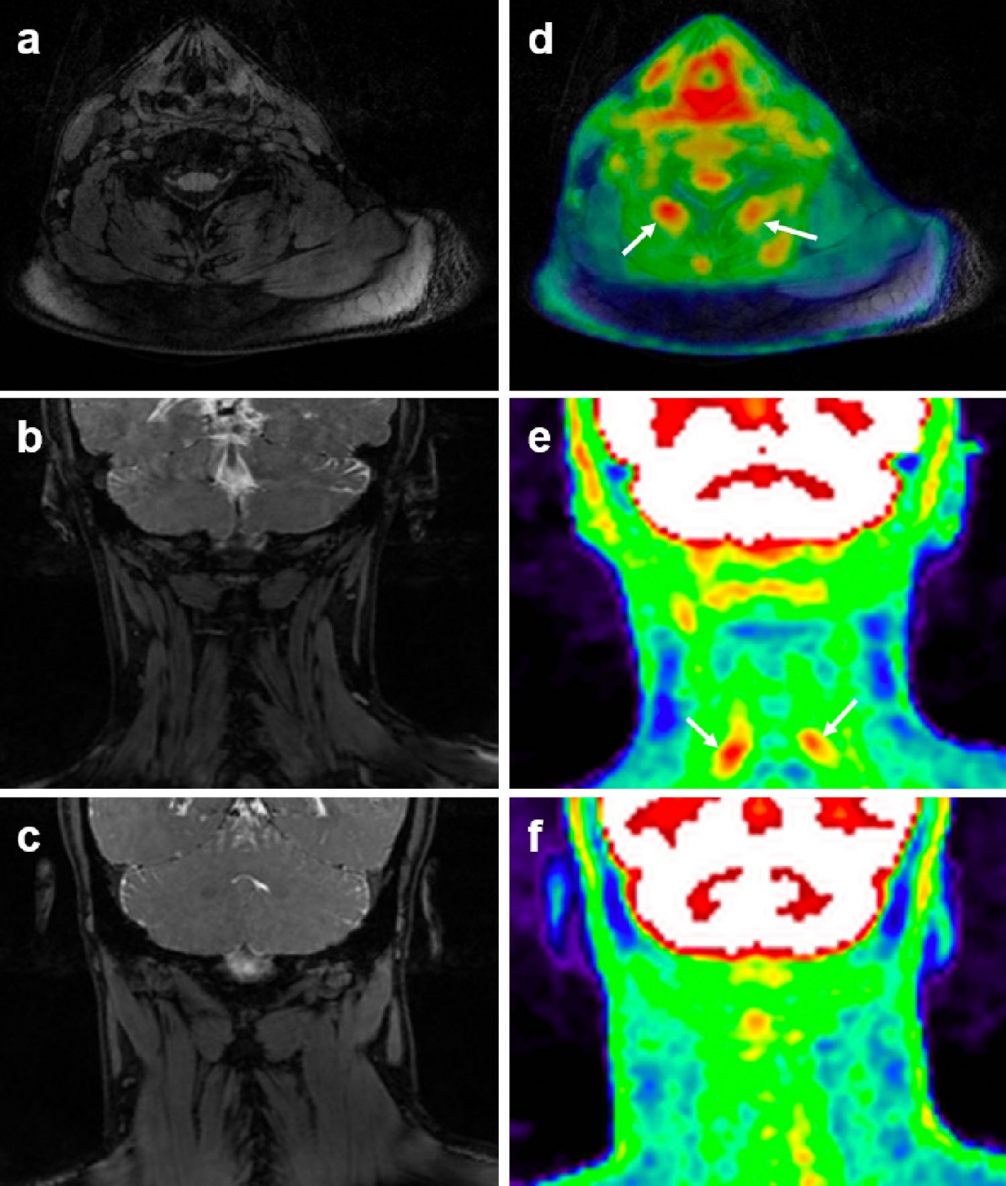
Increased [18F]FDG uptake in cervical paraspinal muscles of patient with suspected cerebrospinal fluid leak**.** Axial MRI at C6 (**a**) appears normal, while the corresponding axial PET/MRI (**d**) shows increased [18F]FDG uptake in paraspinal muscles (arrows, mean SUV_max_ = 2.24) of a CSF-leak patient presenting with head pain. The same pattern is also shown in coronal MRI (**b**) and PET/MRI (**e**). The corresponding coronal MRI and PET/MRI images from a healthy control subject is displayed in (**c**) and (**f**), respectively. No abnormal focal uptake of [18F]FDG as high as those in the patient case is observed in the asymptomatic subject (mean SUV_max_ = 1.38).

**Figure 2:**
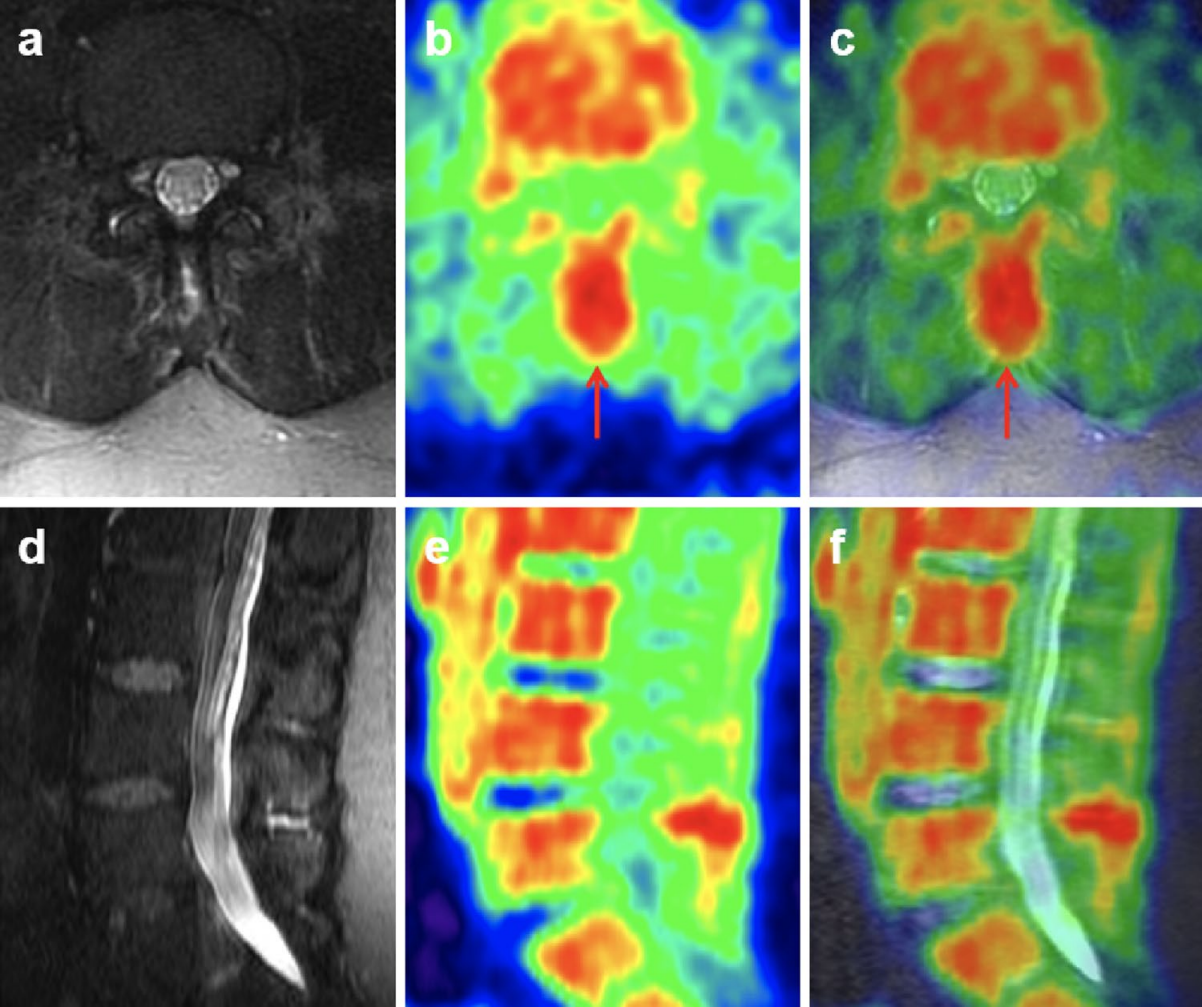
[18F]FDG PET/MRI abnormalities at L4-5 at level of interspinous ligament of patient with suspected cerebrospinal fluid leak, not observed on conventional MRI. High resolution axial MSDE-UBE MRI (**a**) shows increased signal in the vicinity of the interspinous ligament and paraspinal muscles, which was not observed in the original conventional lumbar spine MRI the patient had recently before this PET/MRI. Axial [18F]FDG PET (**b**) and PET/MRI (**c**) shows increased radiotracer uptake posterior to the spinal canal in the region of the interspinous ligament (red arrow, SUV_max_ 3.25). Sagittal reformats of the MSDE-CUBE MRI, PET, and PET/MRI are shown in (**d**), (**e**), and (**f**), respectively to help appreciating the abnormally increased uptake in this level compared to those in other levels.

**Figure 3:**
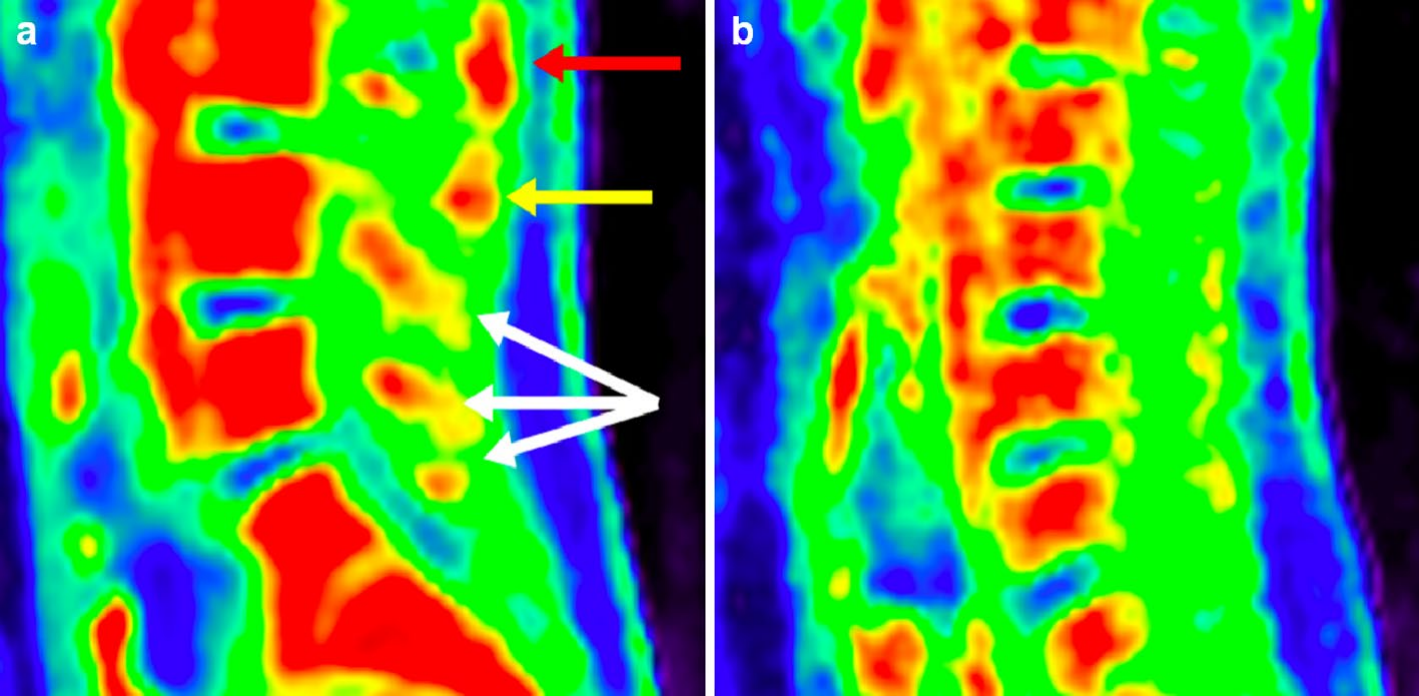
Increased spinal uptake of [18]F-FDG in patient with suspected cerebrospinal fluid leak. Sagittal PET of patient with suspected CSF leak (**a**) shows areas of increased tracer uptake in the region of the interspinous ligament between L1 and L2 (red arrow, SUV_max_ = 2.90), in the region of the interspinous ligament between L2 and L3 (yellow arrow, SUV_max_ = 2.14), and in the area between the spinal cord and vertebral arch of the lower lumbar vertebrae (white arrows, SUV_max_ at L3 = 2.02, SUV_max_ at L4 = 2.22). By comparison, sagittal PET of a healthy control (**b**) at the same level does not show increased uptake in these regions (Mean SUV_max_ between L1 and L2 = 1.23, between L2 and L3 = 1.23, at L3 = 0.98, at L4 = 0.98 for controls). This patient underwent paramedian interlaminar blood patching at L1-2 and L2-3, left transforaminal blood patching at L1-2 and L2-3, and right paramedian caudal fibrin blood patching following the PET/MRI study. The patient reported these patches made him feel better for a week, unlike previous patches that had provided no relief at all.

**Figure 4:**
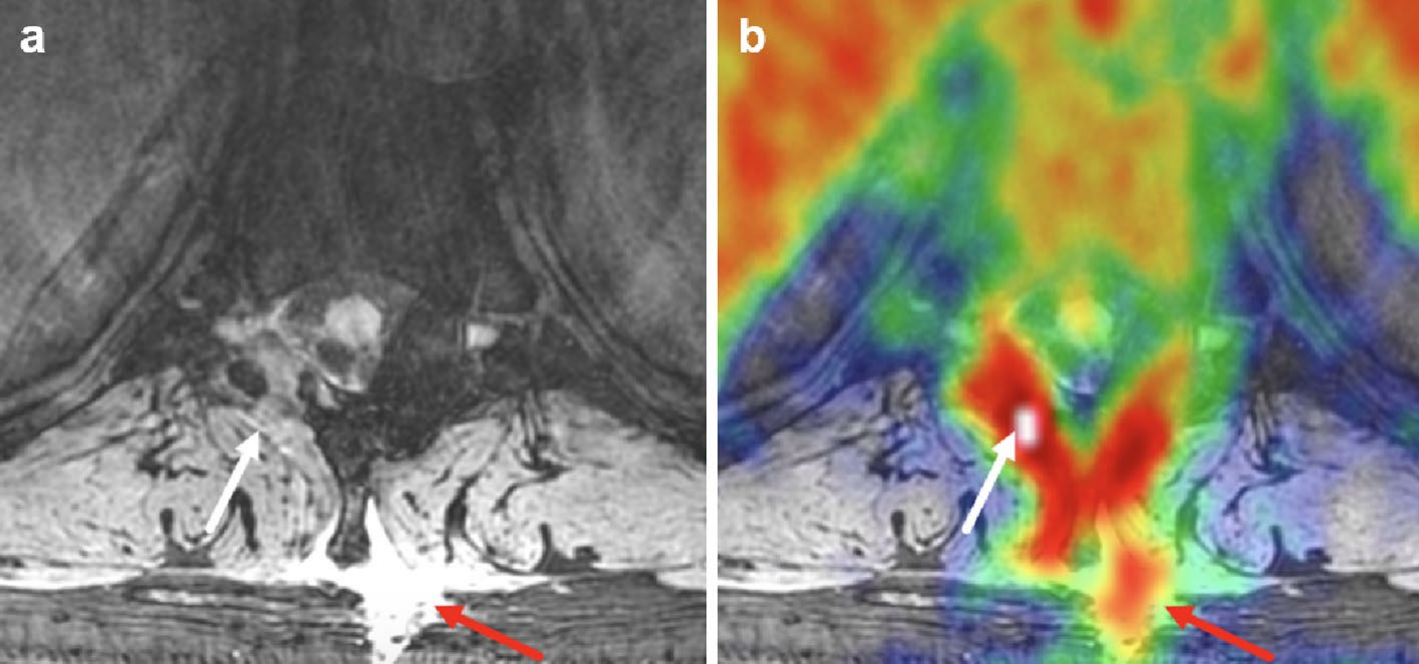
PET/MRI showing abnormalities at the level T10, especially at the site of a right-sided laminotomy, in patient with suspected cerebrospinal fluid leak. Axial DESS MRI (**a**) at T10 level of Patient 6 shows right-sided laminotomy (white arrow) with fluid collection (red arrow). Increased [18F]FDG uptake (white arrow, SUV_max_ = 4.6) is visible on PET/MRI (**b**) in areas corresponding to osseous tissue and neuroforamina. In addition, focal fluid collection is observed around the spinous process, which also has increased [18F]FDG uptake (red arrow, SUV_max_ = 2.6).

Figures 1, 2, 3, and 4 present different cases of abnormally increased [18F]FDG uptake in patients: in cervical paraspinal muscles (Fig. 1), at the level of the interspinous ligament in the lumbar spine (Figs. 2 and 3a), and in areas corresponding to fluid, neuroforamina, and osseous tissue (Fig. 4). The controls did not show areas of focally increased [18F]FDG uptake in these paraspinal regions as shown in an example case of Fig. 3b. Note that the cases in Figs. 1 and 3 show no abnormal MRI signal change, whereas in Fig. 2, increased signal was observed on MSDE-CUBE MRI images in the vicinity of interspinous ligament and paraspinal muscles with high [18F]FDG uptake.

### Blood-patch treatment outcome

Epidural blood-patching was performed on multiple levels of the spine for three patients. Two patients underwent epidural blood-patching on the sites covering abnormalities identified by our PET/MRI study and reported temporary improvement in symptoms significantly greater than previous blood-patches (Table 3). However, the other patient whose blood-patch sites marginally included the PET/MRI abnormalities reported no symptomatic improvement.

**Table 3:** Blood patch treatment information and the treatment outcome.

Patient	Imaging/Treatment date (mm.dd.yy)	Treatment sites	PET/MRI findings and treatment sites congruous?	Symptomatic relief after treatments
Pt.2	02.28.18/05.14.18	Left paramedian L1-3, Right paramedian caudal	Yes	Substantial for 1 week
Pt.4	03.28.18/06.04.18	Right T9-10, L2-3, Caudal	Yes	Substantial for 8-plus weeks
Pt.6	05.29.18/08.24.18	Bilateral L5-S1, Right paramedian L1-2	No	None

## Discussion

In this report, we introduce a novel [18F]FDG PET/MRI approach for detecting the sites of CSF leak. While other nuclear medicine approaches such as radioisotope cisternography have been used to identify CSF leaks^24^, to our knowledge neither [18F]FDG PET nor simultaneous PET/MRI has been used for this purpose until now. In this study, all 6 patients presented significantly higher uptake values of [18F]FDG at various paraspinal structures than those of healthy controls (*P* < 0.05). On the other hand, abnormalities on MRI that might be related to CSF leak were identified in only half of the patients. In a limited number of patients followed for the treatment outcome, the patients whose blood-patch treatment sites covered the abnormalities identified by our method achieved better symptomatic relief than the patient whose treatment marginally included the identified abnormalities.

The proposed approach for the improved detection of CSF leak is based on the observation that extradural CSF may irritate and inflame tissues outside of the nervous system^22^. This CSF-induced irritation is reflected clinically in studies of patients with chronic CSF leaks following accidental dural puncture, who report much higher rates of spinal pain at the site of CSF leak than controls without dural puncture^10,25^. Lesions with active inflammatory responses can have increased metabolism and energy requirements, becoming more glucose-avid than normal tissues^26^. [18F]FDG PET is a highly sensitive imaging modality of glucose metabolism, and has demonstrated its effectiveness in detection of such hypermetabolic inflammation^27–29^. Our results show abnormally increased uptake of [18F]FDG in the paraspinal region of all patients, supporting the feasibility of our approach for the detection of CSF leak. More abnormalities were found with [18F]FDG PET than 3T MRI, which suggests [18F]FDG PET might be better suited than MRI for the detection of early inflammatory changes due to CSF leak. However, our approach may not be feasible for detecting other etiologies without extradural CSF inflammation, such as CSF-venous fistular where CSF escapes the thecal sac directly into adjacent epidural veins.

Unlike the conventional imaging methods for CSF leak diagnosis, our [18F]FDG PET approach is to find the paraspinal tissues potentially affected by CSF leak rather than the tear or puncture through which the CSF leaks. Therefore, using [18F]FDG PET in conjunction with an imaging modality that enables the investigation of the anatomic cause of the leak can create a synergistic effect in accurately specifying the site(s) of CSF leak. MRI is more advantageous than CT for accompanying [18F]FDG PET in that it can provide detailed anatomic examination with no further deposition of ionizing radiation to sensitive areas. The simultaneous acquisition of PET and MRI employed in this study also offers improved image co-registration compared to the conventional PET/CT that sequentially acquires PET and CT, rendering it susceptible to mis-registration due to patient motion between the two scans. Our PET/MRI approach has the additional benefit of potentially discriminating a benign post-operative seroma from an extraspinal collection of CSF. On MRI, they are difficult to differentiate because they are likely to appear isointense to each other. However, on [18F]FDG PET, the benign seroma is expected to have minimal or no [18F]FDG uptake, while the extraspinal CSF collection is significantly [18F]FDG avid, such as the one seen in Patient 6 (Fig. 4). Indeed, a study by Amini et al.^30^ has demonstrated that seromas typically present a thin rim of mildly increased [18F]FDG uptake surrounding a central area of no [18F]FDG uptake in contrast to ‘solid’ or ‘thick-walled’ [18F]FDG avidity of more aggressive lesions such as soft tissue sarcomas.

Our study has a few limitations. More study subjects are needed to determine whether this [18F]FDG PET/MRI approach is overall more sensitive and specific to CSF leaks than other imaging modalities. Uptake of [18F]FDG is relatively non-specific and can be seen in other paraspinal inflammatory processes such as myositis and in musculoskeletal degenerative changes such as Baastrup’s disease^31,32^. Therefore, careful consideration of other etiologies should be considered when abnormal uptake of [18F]FDG is observed in the paraspinal regions. Our early results suggest it could be effective as an additional tool for locating the sites of CSF leak and target sites for epidural patching. Optimizing MRI sequences that can better complement [18F]FDG PET is also necessary to pinpoint the site of CSF leaks. Additionally, post-treatment scans in these same patients will be useful in determining whether a decrease in symptoms correlates with a decrease in [18F]FDG tracer uptake.

## Conclusion

Increased [18F]FDG uptake can be observed on PET in the paraspinal tissues of patients with suspected CSF leak. Our results suggest [18F]FDG PET/MRI is sensitive to abnormalities potentially due to suspected CSF leak, which are not necessarily visible on the standard-of-care diagnostic imaging modalities.

## Methods

### Ethics statement

All experimental protocols in our prospective observational research were approved by the institutional review board (IRB) of the Research Compliance Office, Stanford University, U.S.A. (protocol number IRB-24972). All methods were conducted in accordance with relevant guidelines and regulations.

### Study participants

For the patient group, 4 male and 2 female patients suspected of suffering from CSF leaks were recruited (mean age ± standard deviation: 36.2 ± 8.9 y; age range: 25–54 y). All patients presented with orthostatic headache and previously received standard-of-care imaging methods (including CT myelogram), which did not identify sources of CSF leak. The events identified by the patients as a precipitating event were epidural steroid injection (Patient 1 and 3), traumatic injury (Patient 4 and 5), lumbar puncture (Patient 6), and none (Patient 2). Six healthy controls (mean age ± standard deviation: 28.5 ± 3.3; age range: 22–31 y) were also imaged with the same [18F]FDG PET/MRI protocol used for patients to compare the [18F]FDG uptake between the patient and control groups. Prior to imaging, all participants signed an informed consent form regarding study participation and publishing acquired data and images. All data were acquired in compliance with the Health Insurance Portability and Accountability Act. The clinical trial registration number of our study is NCT03195270 (registration date: 07/22/2017), and the name of the associated clinical trial registry is ClinicalTrials.gov.

### Image acquisition

All subjects were requested to fast for 4 hours before our imaging study to avoid non-specific elevation of the blood glucose level. One hour after a single 10-mCi injection of [18F]FDG, subjects were admitted to a SIGNA PET/MRI scanner (GE Healthcare, Waukesha, WI, U.S.) for imaging. We performed a whole-body PET/MRI scan of subjects, which consisted of 8 to 10 consecutive imaging stations. In each station, 4–8-min simultaneous PET/MRI scan was performed, making the total scan time to be 1 to 1.5 h depending on the patient’s height. Acquired PET and MRI raw data were reconstructed using the algorithms equipped in the scanner^33,34^.

In each station, we conducted following two MRI sequences: 3D axial T1-weighted spoiled gradient-recalled echo sequence with two-point Dixon fat–water separation (TR: 4.6 ms, TE: 1.8 ms, resolution: 1.3 × 1.3 × 3.4 mm, flip angle: 15°) and 2D axial T2-weighted fast-spin-echo sequence with two-point Dixon fat–water separation (TR: 7.6 s TE: 93.3 ms, resolution: 1.5 × 1.5 × 4 mm, echo train length: 15). For one or two imaging stations where we suspected CSF leaks might occur based on the patient’s history, we additionally ran the following two sequences for high-resolution imaging: 3D axial double-echo-in-steady-state (DESS) with water-only excitation (TR: 18.7 ms, TE: 8.1 ms, resolution: 0.8 × 0.8 × 2 mm, flip angle: 30°) and 3D coronal motion-sensitized-driven-equilibrium (MSDE) fast-spin-echo sequence (CUBE) with triple-echo Dixon fat–water separation (TR: 2.5 s, TE: 71.8 ms, resolution: 1.4 × 1.4 × 1.4 mm echo train length: 80). For MRI signal acquisition, a 16-channel head-neck coil, an integrated spine coil, and two 32-channel anterior body array coils were employed.

### Data analysis

Two radiologists reviewed patient PET and MRI images concurrently to find abnormalities that are likely to be related with patients’ symptoms. Abnormal [18F]FDG hotspots were identified as potential lesions and the maximum standardized uptake value (SUV_max_) of [18F]FDG was measured. We used image analysis software (Horos v.3.3.5, 64 bit) for measuring SUV_max_. The lesions on the PET images were categorized into 5 types: paraspinal muscle, interspinous ligament, osseous tissue, neuroforamina, and fluid collection. The SUV_max_ measurements in each lesion type were compared with the SUV_max_ of corresponding areas in the healthy controls using a two-sided Mann–Whitney U-test. We employed this nonparametric test because we could not assume the normality in the measurements from patients and the sample size was small. *P*-value of 0.05 was adopted as a significance level.

### Patient follow-up

After our imaging study, three patients received epidural blood-patch treatments based on the information from the standard-of-care diagnostic methods, without using our imaging findings. Patient 2 had targeted fibrin epidural patches while Patients 4 and 6 had targeted epidural blood patches. When a leak site has been identified, fibrin patches may resolve the symptoms of some patients with CSF leak refractory to epidural patching with blood^35^. The intervals between the imaging and the treatment of these patients were about 2.5 months, 2 months, and 3 months, respectively. We recorded the patient’s qualitative evaluation on the symptomatic improvements (Yes/No) following the treatments in comparison with previous treatments, if possible. We compared the sites of the blood patches with the sites of PET/MRI abnormalities from our study to assess the relevance of detected abnormalities to the symptom based on the patient’s post-treatment evaluation.

## Data Availability

The datasets generated during and/or analyzed during the current study are not publicly available due to the fact we are (1) describing the off-label use of [18F]fluorodeoxyglucose and (2) the data is currently being utilized/analyzed for development of intellectual property, but are available from the corresponding author on reasonable request.
